# Burnout in the Geropsychology Workforce: Results from a National Survey on the Impact of COVID-19

**DOI:** 10.1093/geroni/igaf122.778

**Published:** 2025-12-31

**Authors:** Sophia Geisser, Zoe Geisser, Julia Boyle, Hannah Bashian, Michelle Mlinac

**Affiliations:** University of Alabama, Tuscaloosa, Alabama, United States; SUNY Albany, Albany, New York, United States; VA Boston Healthcare System, Boston, Massachusetts, United States; VA Boston Health Care System, Boston, Massachusetts, United States; VA Boston Healthcare System, Boston, Massachusetts, United States

## Abstract

A geropsychology workforce is critical as the aging population increases. Faculty burnout, exacerbated by COVID-19, poses a significant risk to the growth of the geropsychology field. Faculty and trainees that worked with older adults reported difficulty with pandemic-related mental health challenges, such as an increased demand for services and telehealth platforms and difficulty separating the home and work environment, as they are often interrelated. The Building Bridges team focused on creating training opportunities at the practicum, internship, and fellowship level to foster an interest in working with older adults. However, faculty burnout emerged as a barrier, prompting the development of a Qualtrics survey to explore this. Geropsychology faculty (n = 35) and students/trainees (n = 24) completed an online survey assessing perceptions of burnout and support during the COVID-19 pandemic. Using mixed methods (SPSS for quantitative and NVivo for qualitative analysis), findings revealed significant negative impacts on faculty and training programs, including burnout, fewer and less competent applicants, and worsened professional behaviors (i.e., reduced camaraderie and formality). Despite these challenges, some faculty noted positive student changes, such as improved work-life boundaries (31.4%) and strengthened intention to specialize in Geropsychology (16.7%). Positive adaptations aimed at enhancing training and student support during the pandemic was also highlighted. Recommendations for training programs to address COVID-19 impacts on faculty and trainee wellness and burnout will be discussed. This study emphasizes implications of pandemic-related effects and supports the development and maintenance of the geropsychology workforce.

